# Supplementary material for: Depression, GABA, and Age Correlate with Plasma Levels of Inflammatory Markers
Source: Int J Mol Sci. 2019 Dec 6;20(24):6172. doi: 10.3390/ijms20246172 (PMC6941074; doi:10.3390/ijms20246172)
Supplement: Supplementary file 1 [file ijms-20-06172-s001.pdf]

Table S1: Primers for RT-qPCR

Genes	Forward Primer Sequence	Reverse Primer Sequence	Amplicon Size (bp)
**Endogenous control**			
TBP	GAGCTGTGATGTGAAGTTTCC	TCTGGGTTTGATCATTCTGTAG	117
IPO8	GCAAAGGAAGGGGAATTGAT	CGAAGCTCACTAGTTTTGACCC	91
**19 $GABA_A$ receptor subunit genes**			
GABRA1 (α1)	GTCACCAGTTTCGGACCCG	AACCGGAGGACTGTCATAGGT	119
GABRA2 (α2)	GTTCAAGCTGAATGCCCAAT	ACCTAGAGCCATCAGGAGCA	160
GABRA3 (α3)	CAACTTGTTTCAGTTCATTCATCCTT	CTTGTTTGTGTGATTATCATCTTCTTAGG	102
GABRA4 (α4)	TTGGGGGTCCTGTTACAGAAG	TCTGCCTGAAGAACACATCCA	105
GABRA5 (α5)	TTGGATGGCTACGACAACAGA	GTCCTCACCTGAGTGATGCG	62
GABRA6 (α6)	ACCCACAGTGACAATATCAAAAGC	GGAGTCAGGATGCAAAACAATCT	67
GABRB1 (β1)	TGCATGTATGATGGATCTTCG	GTGGTATAGCCATAACTTTCGA	80
GABRB1 (β1)	ATTACAATTCTGTCCTGGGTG	CACTGTCGTGATTCCTAGTG	81
GABRB2 (β2)	GCAGAGTGTCAATGACCCTAGT	TGGCAATGTCAATGTTCATCCC	137
GABRB3 (β3)	CAAGCTGTTGAAAGGCTACGA	ACTTCGGAAACCATGTCGATG	108
GABRG1 (γ1)	CCTTTTCTTCTGCGGAGTCAA	CATCTGCCTTATCAACACAGTTTCC	91
GABRG2 (γ2)	CACAGAAAATGACGGTGTGG	TCACCCTCAGGAACTTTTGG	136
GABRG3 (γ3)	AACCAACCACCACGAAGAAGA	CCTCATGTCCAGGAGGGAAT	113
GABRD (δ)	CTTTGCTCATTTCAACGCC	TTCCTCACGTCCATCTCTG	86
GABRE (ε)	ACAGGAGTGAGCAACAAAACTG	TGAAAGGCAACATAGCCAAA	107
GABRQ (θ)	CCAGGGTGACAATTGGCTTAA	CCCGCAGATGTGAGTCGAT	63
GABRP (π)	CAATTTTGGTGGAGAACCCG	GCTGTCGGAGGTATATGGTG	110
GABRR1 (ρ1)	AAAGGCAGGCCCCAAAGA	TCAGAATTGGGCTGACTTGCT	70
GABRR2 (ρ2)	TACAGCATGAGGATTACGGT	CAAAGAACAGGTCTGGGAG	81
GABRR3 (ρ3)	TGATGCTTTCATGGGTTTCA	CGCTCACAGCAGTGATGATT	111
**2 $GABA_B$ receptor subunit genes**			
GABBR1 (GABA-B1)	TGGCATGGACGCTTATCGA	GATCATCCTTGGTGCTGTCATAGT	78
GABBR2 (GABA-B2)	GAGTCCACGCCATCTTCAAAAAT	TCAGGATACACAGGTCGATCAGC	108
**6 Chloride transporter genes**			
SLC12A2 (NKCC1)	TGGGTCAAGCTGGAATAGGTC	ACCAAATTCTGGCCCTAGACTT	161

SLC12A1 (NKCC2)	TCAGGAGATTTGGAGGATCCC	ACCCCTAAGTAGGCAACAGTG	86
SLC12A4 (KCC1)	CCTCCCGTGTTTCCGGTATG	CAGGAGTCGGTCGTAAGGTTG	155
SLC12A5 (KCC2)	GGAAGGAAATGAGACGGTGA	TCCCACTCCTCTCCACAATC	200
SLC12A6 (KCC3)	GGATGTCATCGAGGACCTGAG	TCGAGCTTTCTTATGTCCGTC	82
SLC12A7 (KCC4)	ATCTACTTCCCTTCCGTGACC	TCTGTGCATCCTTGAGGTCC	70

Table S2: Demographic characteristic of all study participants

Control blood donors		Patient		
**Age (Years)**	**Sex**	**Age (Years)**	**Sex**	**MADRS-S score**
55	Woman	75	Man	27
52	Man	55	Woman	48
34	Woman	42	Man	19
47	Man	38	Woman	46
47	Man	63	Woman	35
55	Woman	23	Man	21
48	Man	46	Woman	34
67	Woman	23	Woman	39
56	Man	40	Woman	30
24	Man	30	Man	36
58	Woman	21	Woman	28
67	Woman	45	Woman	33
59	Man	59	Man	42
36	Woman	53	Woman	30
33	Man	55	Man	43
35	Woman	49	Woman	30
42	Woman	33	Man	38
72	Man	74	Man	42
45	Man	48	Man	35
62	Woman	34	Woman	30
49	Woman	27	Man	39
29	Woman	49	Man	31
25	Woman	18	Man	28
44	Woman	40	Woman	24
24	Man	59	Woman	37
25	Man			

The contingency of sex equality between the two groups was accessed by Fisher's exact test ($p = 0.99$) and age was accessed by non-parametric Mann-Whitney test ($p = 0.61$).

Table S3: List of biomarkers in Olink's inflammation panel

Adenosine Deaminase (ADA)
Artemin (ARTN)
Axin-1 (AXIN1)
Beta-nerve growth factor (Beta-NGF)
C-C motif chemokine 19 (CCL19)
C-C motif chemokine 20 (CCL20)
C-C motif chemokine 23 (CCL23)
C-C motif chemokine 25 (CCL25)
C-C motif chemokine 28 (CCL28)
C-C motif chemokine 3 (CCL3 / MIP-1 alpha)
C-C motif chemokine 4 (CCL4)
C-X-C motif chemokine 1 (CXCL1)
C-X-C motif chemokine 10 (CXCL10)
C-X-C motif chemokine 11 (CXCL11)
C-X-C motif chemokine 5 (CXCL5)
C-X-C motif chemokine 6 (CXCL6)
C-X-C motif chemokine 9 (CXCL9)
Caspase-8 (CASP-8)
CD40L receptor (CD40)
CUB domain-containing protein 1 (CDCP1)
Cystatin D (CST5)
Delta and Notch-like epidermal growth factor-related receptor (DNER)
Eotaxin (CCL11)
Eukaryotic translation initiation factor 4E-binding protein 1 (4E-BP1)
Fibroblast growth factor 19 (FGF-19)
Fibroblast growth factor 21 (FGF21)
Fibroblast growth factor 23 (FGF-23)
Fibroblast growth factor 5 (FGF-5)
Fms-related tyrosine kinase 3 ligand (Flt3L)
Fractalkine (CX3CL1)
Glial cell line-derived neurotrophic factor (GDNF)
Hepatocyte growth factor (HGF)
Interferon gamma (IFN-gamma)
Interleukin-1 alpha (IL-1 alpha)
Interleukin-10 (IL10)
Interleukin-10 receptor subunit alpha (IL-10RA)
Interleukin-10 receptor subunit beta (IL-10RB)
Interleukin-12 subunit beta (IL-12B)
Interleukin-13 (IL-13)
Interleukin-15 receptor subunit alpha (IL-15RA)
Interleukin-17A (IL-17A)
Interleukin-17C (IL-17C)
Interleukin-18 (IL-18)
Interleukin-18 receptor 1 (IL-18R1)
Interleukin-2 (IL-2)
Interleukin-2 receptor subunit beta (IL-2RB)
Interleukin-20 (IL-20)
Interleukin-20 receptor subunit alpha (IL-20RA)

Interleukin-22 receptor subunit alpha-1 (IL-22 RA1)
Interleukin-24 (IL-24)
Interleukin-33 (IL-33)
Interleukin-4 (IL-4)
Interleukin-5 (IL5)
Interleukin-6 (IL6)
Interleukin-7 (IL-7)
Interleukin-8 (IL-8)
Latency-associated peptide transforming growth factor beta-1 (LAP TGF-beta-1)
Leukemia inhibitory factor (LIF)
Leukemia inhibitory factor receptor (LIF-R)
Macrophage colony-stimulating factor 1 (CSF-1)
Matrix metalloproteinase-1 (MMP-1)
Matrix metalloproteinase-10 (MMP-10)
Monocyte chemotactic protein 1 (MCP-1)
Monocyte chemotactic protein 2 (MCP-2)
Monocyte chemotactic protein 3 (MCP-3)
Monocyte chemotactic protein 4 (MCP-4)
Natural killer cell receptor 2B4 (CD244)
Neurotrophin-3 (NT-3)
Neurturin (NRTN)
Oncostatin-M (OSM)
Osteoprotegerin (OPG)
Programmed cell death 1 ligand 1 (PD-L1)
Protein S100-A12 (EN-RAGE)
Signaling lymphocytic activation molecule (SLAMF1)
SIR2-like protein 2 (SIRT2)
STAM-binding protein (STAMBP)
Stem cell factor (SCF)
Sulfotransferase 1A1 (ST1A1)
T cell surface glycoprotein CD6 isoform (CD6)
T-cell surface glycoprotein CD5 (CD5)
Thymic stromal lymphopoietin (TSLP)
TNF-beta (TNFB)
TNF-related activation-induced cytokine (TRANCE)
TNF-related apoptosis-inducing ligand (TRAIL)
Transforming growth factor alpha (TGF-alpha)
Tumor necrosis factor (Ligand) superfamily, member 12 (TWEAK)
Tumor necrosis factor (TNF)
Tumor necrosis factor ligand superfamily member 14 (TNFSF14)
Tumor necrosis factor receptor superfamily member 9 (TNFRSF9)
Urokinase-type plasminogen activator (uPA)
Vascular endothelial growth factor A (VEGF-A)

<u>Table S4</u>: The percentage of plasma samples from CBD and patients groups together that have expressed the particular cytokine/marker

	% Expression
**MMP-1**	100
**MCP-1**	100
**CD40**	100
**uPA**	100
**SCF**	100
**OPG**	100
**VEGF-A**	100
**CXCL5**	100
**CCL23**	100
**TWEAK**	100
**CCL19**	100
**Flt3L**	100
**CCL11**	100
**IL-18**	100
**MCP-2**	100
**CXCL11**	100
**CXCL1**	100
**TRAIL**	100
**CXCL10**	100
**DNER**	100
**FGF-19**	100
**CSF-1**	100
**CXCL6**	100
**HGF**	100
**4E-BP1**	100
**IL-18R1**	100
**CCL4**	100
**CXCL9**	100
**LAP TGF-beta-1**	100
**IL-10RB**	100
**IL-10RA**	33
**IL-8**	100
**CST5**	100
**CD244**	100
**TNFRSF9**	100
**MMP-10**	100
**CD5**	100
**AXIN1**	100
**CCL25**	100
**CX3CL1**	100
**IL-12B**	100

**FGF-21**	100
**STAMPB**	100
**TRANCE**	100
**CD6**	100
**CCL20**	100
**ADA**	100
**TNFSF14**	100
**TNFB**	100
**LIF-R**	100
**SIRT2**	96
**PD-L1**	100
**MIP-1 alpha**	100
**IL-6**	43
**IL-7**	100
**OSM**	100
**ST1A1**	88
**MCP-4**	100
**CASP-8**	96
**SLAMF1**	86
**TGF-alpha**	100
**FGF-23**	100
**CDCP1**	100
**Beta-NGF**	84
**CCL28**	100
**EN-RAGE**	88
**NT-3**	70

Table S5: Correlation of expression level of cytokines in plasma with age or GABA concentration in CBD and patients

CBD: Correlation with Age

Cytokine	rho (R)	P value	summary	Adjusted P value	summary
CDCP1	0.8351	<0.0001	****	0.005	**
FGF-21	0.5038	0.0142	*	0.035	*
IL-8	0.6299	0.0013	**	0.010	*
CXCL9	0.5656	0.0049	**	0.020	*
HGF	0.4994	0.0153	*	0.040	*
CXCL10	0.5335	0.0088	**	0.030	*
Flt3L	0.5701	0.0045	**	0.015	*
VEGF-A	0.4208	0.0456	*	0.050	*
OPG	0.44	0.0356	*	0.045	*
MMP-1	0.5404	0.0078	**	0.025	*
**CBD: Correlation with GABA**					
LIF-R	0.4704	0.0421	*	0.025	*
ST1A1	-0.4874	0.0402	*	0.05	*

Patients: Correlation with Age

Cytokine	rho (R)	P value	summary	Adjusted P value	summary
EN-RAGE	0.4867	0.0216	*	0.0405	*
TGF-alpha	0.554	0.0041	**	0.0190	*
SLAMF1	0.6552	0.0013	**	0.0048	**
MCP-4	0.5382	0.0055	**	0.0262	*
OSM	0.5667	0.0031	**	0.0167	*
IL-7	0.4435	0.0264	*	0.0452	*
IL-12B	-0.4258	0.0338	*	0.0476	*
CX3CL1	0.4497	0.0241	*	0.0429	*
CST5	0.636	0.0006	***	0.0024	**
IL-8	0.5713	0.0029	**	0.0119	*
LAP TGF-beta-1	0.4608	0.0204	*	0.0357	*
CXCL9	0.4605	0.0205	*	0.0381	*
HGF	0.5702	0.0029	**	0.0143	*
CXCL1	0.5494	0.0044	**	0.0214	*
MCP-2	0.5074	0.0096	**	0.0286	*
CCL11	0.5767	0.0025	**	0.0095	**
CCL23	0.5413	0.0052	**	0.0238	*
VEGF-A	0.4793	0.0153	*	0.0333	*
OPG	0.594	0.0017	**	0.0071	**
MCP-1	0.5063	0.0098	**	0.0310	*
MMP-1	0.4142	0.0395	*	0.0500	*

**Patients: Correlation with MADRS-S score**					
IL-18	-0.4832	0.0168	*	0.017	*

The p values for the spearman correlation before and after the Bejamini and Hochberg correction procedure for false discovery rate of 5% are indicated as p value and adjusted p value, respectively.

Table S6: The percentage of supernatant samples of stimulated PBMC from patients that have expressed the particular cytokine/marker

	% Expression
**IL-8**	100
**TNF**	100
**CCL4**	100
**CXCL10**	100
**MMP-1**	100
**MCP-1**	100
**CXCL1**	100
**MCP-2**	100
**CXCL5**	100
**MIP-1 alpha**	100
**TNFRSF9**	100
**OSM**	100
**TNFB**	100
**CXCL9**	100
**LIF**	100
**IL-17A**	100
**IL-6**	100
**CCL20**	100
**IFN-gamma**	100
**4E-BP1**	100
**IL-1 alpha**	100
**CD40**	100
**MCP-3**	100
**uPA**	100
**CSF-1**	100
**TNFSF14**	100
**IL-12B**	100
**IL-13**	100
**CD5**	100
**Flt3L**	100
**IL-10**	100
**CASP-8**	100
**ADA**	100
**CXCL6**	100
**IL-18R1**	100
**CCL19**	100
**TRANCE**	100
**MMP-10**	100
**LAP TGF-beta-1**	100
**OPG**	100

**IL-2**	86
**STAMPB**	100
**PD-L1**	100
**VEGF-A**	100
**CD6**	100
**TWEAK**	100
**CXCL11**	100
**TGF-alpha**	100
**TRAIL**	100
**HGF**	100
**IL-4**	100
**SIRT2**	100
**IL-24**	93
**EN-RAGE**	100
**CCL23**	100
**IL-18**	100
**IL-5**	83
**CD244**	100
**DNER**	100
**CDCP1**	100
**AXIN1**	100
**SLAMF1**	89
**SCF**	100
**IL-10RA**	85
**ST1A1**	100
**IL-2RB**	63
**IL-15RA**	70
**CCL28**	61

Table S7: GABA treatment alters markers released by PBMCs from patients

GABA 100 nM	
**Markers**	**P values**
**AXIN1**	0.016
**TNFRSF9**	0.015
**VEGF-A**	0.016
**IL-1α**	0.015

GABA 500 nM	
**Markers**	**P values**
**CD244**	0.016
**IL-13**	0.016
**HGF**	0.015
**VEGF-A**	0.003

Table S8: **Shapiro-Wilk normality test**

	P value	Passed normality test?	Test performed
**GABA receptor subunits**			
ρ2	<0.0001	No	Mann-Whitney
GABA-B1	0.0027	No	Mann-Whitney
**Chloride transporters**			
NKCC1	0.088	Yes	Unpaired T-test
KCC1	<0.0001	No	Mann-Whitney
KCC3	0.0004	No	Mann-Whitney
KCC4	<0.0001	No	Mann-Whitney
